# Whole Transcriptome Analysis of Hypothalamus in Mice during Short-Term Starvation

**DOI:** 10.3390/ijms24043204

**Published:** 2023-02-06

**Authors:** Eun-Young Oh, Byong Seo Park, Hye Rim Yang, Ho Gyun Lee, Thai Hien Tu, Sunggu Yang, Mi-Ryung Han, Jae Geun Kim

**Affiliations:** 1 Division of Life Sciences, College of Life Sciences and Bioengineering, Incheon National University, Incheon 22012, Republic of Korea; 2 Department of Nano-Bioengineering, Incheon National University, Incheon 22012, Republic of Korea

**Keywords:** appetite, RNA-seq, hypothalamus, secretory factor, food deprivation, energy homeostasis

## Abstract

Molecular profiling of the hypothalamus in response to metabolic shifts is a critical cue to better understand the principle of the central control of whole-body energy metabolism. The transcriptional responses of the rodent hypothalamus to short-term calorie restriction have been documented. However, studies on the identification of hypothalamic secretory factors that potentially contribute to the control of appetite are lacking. In this study, we analyzed the differential expression of hypothalamic genes and compared the selected secretory factors from the fasted mice with those of fed control mice using bulk RNA-sequencing. We verified seven secretory genes that were significantly altered in the hypothalamus of fasted mice. In addition, we determined the response of secretory genes in cultured hypothalamic cells to treatment with ghrelin and leptin. The current study provides further insights into the neuronal response to food restriction at the molecular level and may be useful for understanding the hypothalamic control of appetite.

## 1. Introduction

A great deal of attention has been paid over the last few decades to identifying the function of the hypothalamus in controlling a wide range of behaviors and physiological adaptions, including reproduction, the circadian rhythm, social behaviors, and multiple-body homeostasis [1,2,3,4,5,6,7,8]. The circuit activity of hypothalamic neurons, in particular, governs the whole-body energy homeostasis by driving the homeostatic behaviors, including energy intake and expenditure, and thus, the perturbation of normal hypothalamic function leads to the development of metabolic disorders, such as obesity and diabetes [9,10]. In line with these aspects, studies on obesity patients and rodent models have revealed that abnormalities in appetite regulation are primarily triggered by impairment of the hypothalamic neurocircuitry [10,11]. Based on these clinically significant findings, intensive studies have been conducted to verify the hypothalamic control of appetite by identifying the molecular mediators in hypothalamic cells and the biochemical components derived from metabolically active peripheral organs. Using tissue specimens from the entire hypothalamus [12] or arcuate nucleus (Arc) [13,14], gene expression profiling in response to short-term calorie restriction has previously been reported using microarray methods, a hybridization-based technology that is commonly used for gene expression profiling. RNA-sequencing (RNA-seq) has recently emerged as a viable alternative for gene expression profiling and has been used for the transcriptome profiling of living organisms. Notably, it has contributed to significant progress in disease diagnosis and obtaining genetic information that can predict disease onset. Although transcriptome profiling of the hypothalamus has been previously performed on some animal models [15,16,17,18], information regarding the hypothalamic mediators that control energy intake and expenditure is insufficient. Particularly, profiling of hypothalamic-specific gene expression associated with appetite regulation has not yet been performed using an RNA-seq technique. In the current study, we performed RNA-seq experiments using the total hypothalamus and hippocampus of young adult male mice after overnight food deprivation. In this study, we aimed to determine the hypothalamus-specific genetic alterations associated with appetite regulation by comparing overnight-fasted and fed control mice using the RNA-seq technique. We then explored the hypothalamus-specific secretory-related genes among the differentially expressed genes (DEGs) and validated their responses to metabolic hormones, including leptin and ghrelin, in a cultured hypothalamic cell line.

## 2. Results

### 2.1. Differentially Expressed Genes

**Figure 1:**
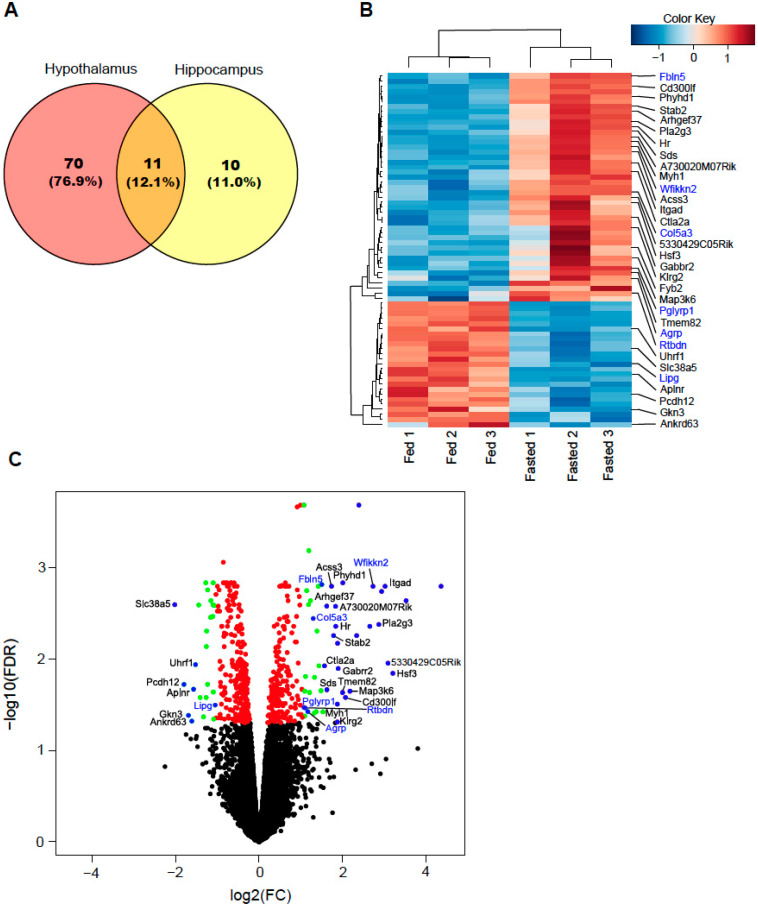
Overview of differential gene expression analysis between fed and fasted mice. (**A**) Venn diagram showing the number of overlapping genes between the hypothalamus and hippocampus regions in genes with false discovery rate (FDR) ≤ 0.05 and absolute log2 fold change (abs(log2FC)) > 1.0. (**B**) Heatmap demonstrating 70 differentially expressed genes (DEGs) with FDR ≤ 0.05 and abs(log2FC) > 1.0 in only hypothalamus, excluding 11 common genes between the hypothalamus and hippocampus. The genes annotated to the right of the heatmap are those with FDR ≤ 0.05 and abs(log2FC) > 1.5, and the blue color text represents the secretory-related gene. (**C**) Volcano plot depicting DEGs and distribution in hypothalamus compared with those of fed mice. The x-axis is log2FC of individual genes and y-axis is the negative logarithm of their FDR to base 10(−log10(FDR)). The black dots indicate genes with FDR > 0.05. The red dots indicate genes with FDR ≤ 0.05 and abs(log2FC) ≤ 1.0. The green dots indicate DEGs with FDR ≤ 0.05 and abs(log2FC) > 1.0 in fasted mice compared with those of fed mice. The blue dots indicate genes identified with FDR ≤ 0.05 and abs(log2FC) > 1.5, and secretory-related genes in fasted mice compared with those of fed mice. The blue color text represents the secretory-related gene.

In order to select the hypothalamus-specific genes that are potentially associated with whole-body energy metabolism, we obtained the profiling results from both the hypothalamus and hippocampus by using the RNA-seq technique. DEGs in the hypothalamus and hippocampus were compared using the edgeR R package. A total of 81 and 21 genes of the hypothalamus and hippocampus, respectively, were differentially expressed, with a false discovery rate (FDR) ≤ 0.05 and logarithm of fold change (log2FC) > 1.0 or <−1.0 in fasted mice compared with those of fed mice (Figure 1A, Tables S1–S3). Among these genes, 11 were detected in both the hypothalamus and hippocampus (Figure 1A, Table S3). Of the 70 hypothalamus-specific genes, 45 were significantly upregulated and 25 were significantly downregulated in fasted mice when compared with those of fed mice (FDR ≤ 0.05, abs(log2FC) >1.0, Table S1). Of the 10 hippocampus-specific genes, 7 significantly upregulated genes and 3 significantly downregulated genes were identified in fasted mice compared to those of fed mice (Table S2). With more stringent criteria (cut-off FDR ≤ 0.05 and abs(log2FC) > 1.5), we found 32 genes from the 70 hypothalamus-specific genes (Table 1). In the hypothalamus of fasted mice, secretory-related genes WAP, follistatin/kazal, immunoglobulin, kunitz, and netrin domain containing 2 (*Wfikkn2*) (log2FC = 2.73, FDR = 0.002), fibulin 5 (*Fbln5*) (log2FC = 1.51, FDR = 0.002); collagen type V alpha 3 (*Col5a3*) (log2FC = 1.14, FDR = 0.002); peptidoglycan recognition protein 1 (*Pglyrp1*) (log2FC = 1.08, FDR = 0.034); agouti related neuropeptide (*Agrp*) (log2FC = 1.24, FDR = 0.054); and retbindin (*Rtbdn*) (log2FC = 1.17, FDR = 0.038) were associated with upregulation and endothelial lipase (*Lipg*) (log2FC = −1.26, FDR = 0.026) was associated with downregulation in the mouse secretome database [19] when compared with those of fed mice (Figure 1B,C, Table 1). 

### 2.2. Secretory-Related Genes Are Altered in the Hypothalamus of Fasted Mice

**Figure 2:**
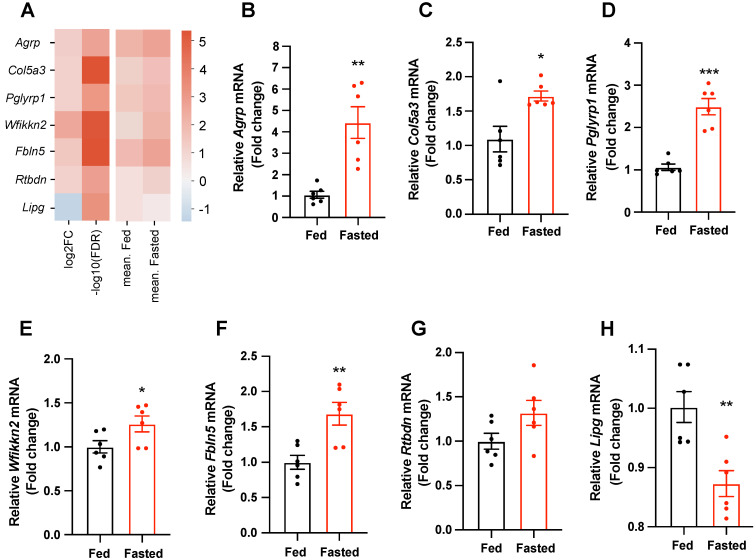
Secretory-related genes are altered in the hypothalamus in response to short-term starvation. Analysis and validation of differentially expressed and secretory-related genes with FDR ≤ 0.05 and abs(log2FC) > 1.0 between the fed and fasted groups. (**A**) Heatmap representing DEGs. These genes encoded secretory signal peptides that were detected by at least three of the four methods (SignalP4, Phobius, TargetP, and WoLF PSORT) and were annotated to be “secreted” or “extracellular” in the subcellular location from the UniProtKB/Swiss-Prot dataset. Blue represents low expression, and red represents high expression in log2FC. (**B**–**F**) The mRNA expression levels of *Agrp*, *Col5a3*, *Pglyrp1*, *Wfikkn2*, and *Fbln5* were significantly elevated in hypothalamus of the fasted mice compared with those of fed control mice. (**G**) *Rtbdn* mRNA levels tended to be increased in hypothalamus of the fasted mice compared with those of fed control mice. (**H**) The mRNA level of *Lipg* was decreased in hypothalamus of the fasted mice compared with that of fed control mice. *n* = 6 mice/group. Data are presented as the mean ± SEM. * *p* < 0.05, ** *p* < 0.01, *** *p* < 0.001 vs. fed. *p*-values for unpaired comparisons were analyzed by two-tailed Student’s *t*-test.

Since secretory factors derived from hypothalamic cells, including neurons, astrocytes, tanycytes, and microglia, act as chemical messengers that regulate the hypothalamic circuit activity, we explored the mouse secretome database [19] and obtained seven secreted genes whose expressions in the hypothalamus of fasted mice were changed compared with those of fed control mice. *Agrp*, *Col5a3*, *Pglyrp1*, *Wfikkn2*, *Fbln5*, and *Rtbdn* were upregulated, and *Lipg* was downregulated (Figure 2A). To confirm the RNA profiling, we determined the mRNA expression levels of secretory-related genes in the hypothalamus of fed and fasted mice using quantitative polymerase chain reaction (qPCR). In accordance with previous reports, the elevated mRNA expression of *Agrp*, the gene encoding an orexigenic neuropeptide, was observed in the hypothalamus of fasted mice compared with that in fed mice (Figure 2B). Fasted mice indicated an increase in the expression of *Col5a3*, a low-abundance fibrillar collagen; *Pglyrp1*, an antibacterial and pro-inflammatory innate immunity protein; *Wfikkn2*, a protease inhibitor; *Fbln5*, a member of the fibulin protein family; and *Rtbdn*, an extracellular rod-expressed protein, in the hypothalamus compared with those in fed mice (Figure 2C–G). In addition, food deprivation resulted in a decrease in the mRNA level of *Lipg*, a member of the lipoprotein lipase family, in the hypothalamus of fasted mice compared with that of fed mice (Figure 2H). 

### 2.3. Selected Secretory-Related Genes Respond to the Metabolic Hormones

**Figure 3:**
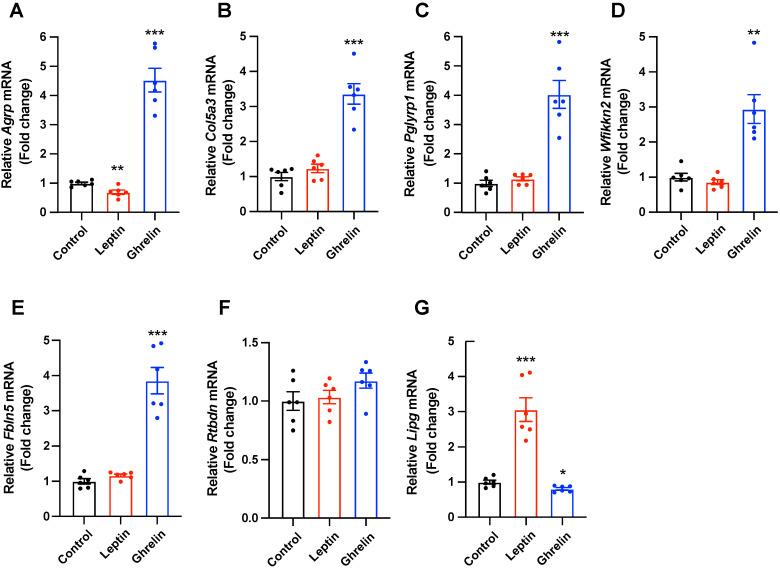
Expression of secretory-related genes was altered by metabolic hormones in mHypoA cells. Quantitative real-time polymerase chain reaction analysis was performed to determine alterations in secretory-related gene expression in the leptin- or ghrelin-treated mHypoA cells. (**A**) The mRNA expression of *Agrp* was decreased by leptin treatment and upregulated by ghrelin treatment. (**B**–**E**) The mRNA levels of *Col5a3*, *Pglyrp1*, *Wfikkn2*, and *Fbln5* were increased by ghrelin treatment, but not altered by leptin treatment. (**F**) *Rtbdn* expression was not altered by leptin or ghrelin treatment. (**G**) The mRNA levels of *Lipg* were increased by leptin treatment and decreased by ghrelin treatment in mHypoA cells. *n* = 6/group. Data are presented as the mean ± SEM. * *p* < 0.05, ** *p* < 0.01, *** *p* < 0.001 vs. control. *p*-values for unpaired comparisons were analyzed by two-tailed Student’s *t*-test.

To verify whether the secretory-related genes selected from the hypothalamus of fasted mice participate in the hypothalamic control of energy metabolism, we evaluated the responsiveness of the seven selected genes to the metabolic hormones, such as leptin, an anorexigenic hormone derived from adipose tissue, and ghrelin, an orexigenic hormone secreted from the gut in the mouse hypothalamic mHypoA cell line. We first confirmed that mRNA levels of *Agrp* were significantly reduced by leptin treatment and drastically elevated by ghrelin treatment (Figure 3A). Further, qPCR data revealed a significant increase in the mRNA expression of *Col5a3*, *Pglyrp1*, *Wfikkn2*, and *Fbln5* in ghrelin-treated mHypoA cells compared with that in the vehicle-treated group. However, no significant alterations in the mRNA levels of these genes were observed in leptin-treated mHypoA cells (Figure 3B–E). The *Rtbdn* mRNA levels were not altered by leptin or ghrelin treatment (Figure 3F). In addition, the mRNA expression of *Lipg* was increased by leptin treatment and decreased by ghrelin treatment in cultured mHypoA cells (Figure 3G). Collectively, these data suggest that selected secretory-related genes can be physiologically involved in hypothalamic functions that integrate metabolic signals.

### 2.4. Enriched Functional Annotation and Canonical Pathway

Enriched functional annotations of 70 hypothalamus-specific genes (FDR ≤ 0.05, abs(log2FC) > 1.0) were obtained using ingenuity pathway analysis (IPA; Table 2). The top 20 enriched functional annotations from IPA are listed in Table 2. Ingestion by mice, hyperphagia, appetite, vascular endothelial cell function, and obesity caused by epididymal fat were among the top functional annotations indicated by this dataset (Table 2). In addition, the top-ranking functional annotation “ingestion by mice”, involving six genes (five upregulated and one downregulated), was associated with digestive system development and function [20,21,22,23,24]. *Agrp* and neuropeptide Y *(Npy)* were functionally annotated in ingestion by mice, hyperphagia, and appetite. These annotations are in accordance with the physiological processes that are known to be involved in food deprivation [25].

As for the canonical pathway analysis results, several pathways associated with a comparison between the hypothalamus of fasted and fed mice were identified: triacylglycerol degradation, gustation pathway, leptin signaling in obesity, ketogenesis, and mevalonate pathway I (Table 3). These pathways include core signaling genes (*Lipg*, *Agtr1a*, *Col5a3*, *Agrp*, *Npy*, and *Hmgcs2*), which are associated with pathways related to fasted mice compared with those of fed mice.

### 2.5. Gene Network Identification

**Figure 4:**
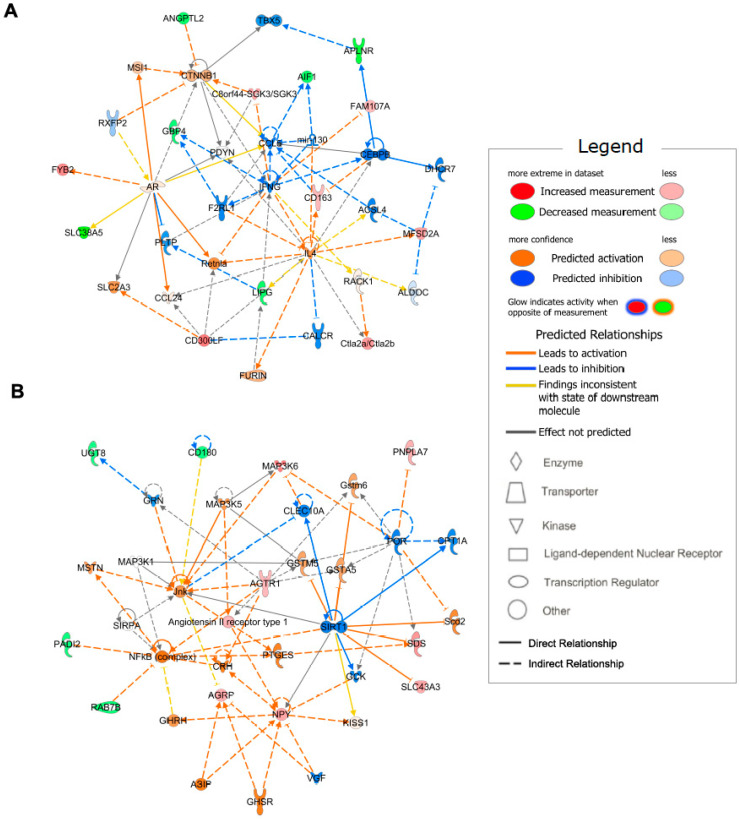
Identification of gene network. Networks obtained using IPA of 70 DEGs in only hypothalamus with FDR ≤ 0.05 and abs(log2FC) > 1.0 in fasted mice compared with those of fed mice. The networks with the highest score are exhibited. Genes in green were downregulated and red were upregulated. (**A**) The network with a score of 23 is associated with lipid metabolism, molecular transport, and small-molecule biochemistry, consisting of *Angptl2*, *Aplnr*, and *Lipg*. (**B**) The network with a score of 19 is associated with digestive system development and function, lipid metabolism, and molecular transport, consisting of *Agrp* and *Npy*.

The top 70 hypothalamus-specific genes were used to identify gene networks. The two networks with the highest scores are depicted in Figure 4. The network with a score of 23, including genes angiopoietin like 2 (*Angptl2*), apelin receptor *(Aplnr)*, and *Lipg*, was associated with lipid metabolism, molecular transport, and small-molecule biochemistry (Figure 4A). In support of this identified network, previous studies have indicated that the lipid metabolism in hypothalamic cells is associated with the physiological and pathological roles of the hypothalamus on whole-body energy metabolism [26,27]. The network with a score of 19 was associated with digestive system development and function, lipid metabolism, and molecular transport, including the *Agrp* and *Npy* genes (Figure 4B). Multiple hormones originating from the digestive system act as afferent inputs to the hypothalamic circuit, thereby controlling homeostatic behaviors [10,11]. Moreover, hypothalamic control of energy metabolism is closely linked to nutrient metabolism controlled by nutrient transporters, including glucose and monocarboxylate transporters [28]. Thus, the identified network depicted in Figure 4B was correlated with selected hypothalamus-specific genes that responded to calorie restriction. Collectively, our network results suggest that the top 70 genes were associated with various cellular processes connected to the metabolic functions of hypothalamic cells.

## 3. Discussion

Energy homeostasis is a critical biological event for maintaining life at the cellular and physiological levels. Thus, it has long been studied to understand the underlying mechanisms of biological processes in a variety of organisms and to develop strategies that can be applied to treat human diseases. In a state of energy deficiency, multiple molecular and biochemical processes occur in various organs. Catabolic processes, in particular, are activated to maintain cellular energy homeostasis [29]. In line with these findings, studies have indicated that cellular and molecular responses coupled with altered energy status in metabolic organs are responsible for the control of whole-body energy metabolism [30]. The hypothalamus is a central unit that governs the systemic regulation of energy balance by integrating afferent inputs derived from peripheral organs and other brain areas that are metabolically involved [10]. Therefore, the responsiveness of hypothalamic cells drives homeostatic behaviors, including energy intake, energy expenditure, and physical activity [31]. In the present study, we performed RNA-seq experiments with the hypothalamus and hippocampus extracted from fed and fasted mice and proposed novel molecular mediators that potentially participate in appetite regulation, using the profiling data of hypothalamus-specific mRNA in a model of short-term calorie restriction. We confirmed the alteration of genes previously defined as appetite regulators, including those that encode hypothalamic neuropeptides, such as Agrp and Npy. The profiling results revealed that 81 genes were altered in the hypothalamus and 21 in the hippocampus under overnight fasting conditions, suggesting that the hypothalamus is more metabolically active than the hippocampus. The analyzed data also indicated that 11 genes were commonly altered in both the hypothalamus and hippocampus, and 70 genes were only altered in the hypothalamus in response to overnight fasting. Among the 45 upregulated genes, we identified the ones that encode metabolic enzymes, such as Hmgcs2, which is involved in the synthesis of ketone bodies. In accordance with these findings, our previous studies indicated that the hunger-promoting condition triggered by fasting led to an increase in ketone bodies and a decrease in lactate in the hypothalamus, while mice retaining anorexigenic responses to lipopolysaccharide treatment revealed a contradicting pattern in the circulating levels of ketone bodies and lactate [32,33]. Circulating factors derived from peripheral organs, such as fat, muscle, and liver, dynamically act as signal messengers that propagate information on the current status of energy availability to integrating centers, such as the hypothalamus. In support of this evidence, hypothalamic neurons strongly express receptors of metabolic hormones, and impairment of these receptors in hypothalamic neurons leads to metabolic abnormalities. Given that hypothalamic neurons also release a variety of signal messengers that trigger homeostatic behaviors, identifying novel factors secreted from hypothalamic cells to better understand the hypothalamic control of whole-body metabolism is significant. Among the 70 hypothalamic genes that responded to short-term calorie restriction, we selected seven secretory-related genes, including *Fbln5*, *Col5a3*, *Pglyrp1*, *Wfikkn2*, *Rtbdn*, *Lipg*, and *Agrp*. Notably, previous studies have identified the roles of Wfikkn2, a protease inhibitor, involved in lipid metabolism in the adipose tissue and brain [34]. In addition, *Lipg,* an enzyme involved in lipid accumulation, was found among the 25 downregulated genes. Since lipid metabolism in hypothalamic cells is regarded as an effective cellular event for maintaining whole-body energy homeostasis, hypothalamic *Wfikkn* and *Lipg* may play a role in the control of energy metabolism. Pglyrp1 is involved in anti- and pro-inflammatory homeostasis and glucose metabolism. Notably, long-term calorie restriction prevents inflammatory responses, which is a possible reason for the beneficial effects of calorie restriction [35]. Our profiling result indicating elevated *Pglyrp1* expression in the hypothalamus under fasting conditions suggests that secreted hypothalamic Pglyrp1 could be associated with cellular homeostatic responses for adjusting the inflammatory tone during starvation. These previous findings and our profiling results suggest that the newly identified secretory-related factors induced by short-term calorie restriction impact the hypothalamic control of energy metabolism. The metabolic functions of the selected secretory-related genes have not yet been unveiled. Therefore, whether they participate in the regulation of energy metabolism governed by the hypothalamic circuit is worth investigating. To further verify the physiological involvement of the selected secretory-related genes in controlling homeostatic behaviors coupled with whole-body energy metabolism, we performed experiments that confirmed the expression patterns of the seven selected secretory-related factors in cultured hypothalamic cells in response to treatment with leptin, an anorexigenic hormone derived from adipose tissues, and ghrelin, an orexigenic hormone originating from the gut. Notably, four of the five selected secretory-related genes, which were upregulated under starvation conditions, positively responded to ghrelin treatment, but not to leptin treatment. In line with these observations, hypothalamic *Lipg* mRNA levels were strongly elevated by leptin treatment and marginally reduced by ghrelin treatment. These observations further suggest that hypothalamic secretory-related factors that are released in increased quantities in response to starvation are oriented toward orexigenic responses during energy-deficit conditions, and hypothalamic *Lipg* mediates the orexigenic and anorexigenic signals from the peripheral organs involved in metabolic regulations. However, further studies are needed to clarify whether the selected secretory factors are actively involved in the hypothalamic control of whole-body energy metabolism by utilizing the recombinant proteins or the transgenic animals, in which the selected secretory genes are specifically ablated in the hypothalamic cells. Collectively, the current findings provide useful information for regarding the whole-body energy metabolism governed by the hypothalamus and for establishing clinical strategies for patients with metabolic disorders.

## 4. Materials and Methods

### 4.1. Animals

Eight-week-old male C57BL/6 mice (Dae Han Bio Link, Eumsung, Republic of Korea) were housed in a 12 h light–dark cycle at 25 °C and 55 ± 5% humidity. The mice were allowed access to normal diet and tap water ad libitum. For food deprivation experiments, food was withdrawn for 18 h starting at 5:00 p.m. Mice were sacrificed by decapitation and their hypothalamus and hippocampus were quickly removed for RNA extraction. All animal care and experimental procedures were performed in accordance with a protocol approved by the Institutional Animal Care and Use Committee (IACUC) at the Incheon National University (permission number: INU-2016-001). 

### 4.2. Culture and Treatment of the Cells

Mouse hypothalamic mHypoA cells purchased from CELLutions Biosystems (CELLutions Biosystems Inc., Toronto, ON, Canada) were cultured in Dulbecco’s modified Eagle medium (DMEM) containing high glucose (Gibco BRL, Grand Island, NY, USA) and 10% (*v*/*v*) fetal bovine serum (Gibco BRL) at 37 °C with 5% CO_2_ condition. For the gene expression assay, mHypoA cells were seeded in 3 × 10^5^ cells/well in 6-well plates and attached overnight. After serum starvation for 5 h, the attached cells were treated with leptin (200 ng/well, R&D Systems, Minneapolis, MN, USA) or ghrelin (400 nM/well, R&D Systems) for 1 h and then subjected to RNA extraction. 

### 4.3. Quantitative Real-Time Reverse Transcription-Polymerase Chain Reaction

Total RNA was extracted from the hypothalamus of mice and cultured mHypoA cells according to the Tri-Reagent (Invitrogen, Carlsbad, CA, USA) protocol. First-strand cDNA was synthesized with 2 μg total RNA using a high-capacity cDNA reverse transcription kit (Intron Biotechnology, Seoul, Republic of Korea). The mRNA expression levels were measured using a Bio-Rad CFX 96 Real-Time Detection System (Bio-Rad Laboratories, Hercules, CA, USA) with the SYBR Green Real-time PCR Master Mix Kit (TaKaRa Bio Inc., Foster, CA, USA). The results were analyzed using the CFX Manager software and normalized to the levels of *β-actin*, a housekeeping gene. The primers used were: *Agrp*, F-TGTGTAAGGCTGCACGAGTC and R-GGCAGTAGCAAAAGGCATTG; *Col5a3*, F-CGGGGAGGAGTCTTTTGAG and R-GCCTGAGGGTCTGGAATTAAC; *Pglyrp1*, F-GTGGTGATCTCACACACAGC and R-GTGTGGTCACCCTTGATGTT; *Wfikkn2*, F-GAGTCGACGCGCACACCGCCCTGCCGCGCC and R-GCGAAGCTTGGAGTGCGTTTATTCACCAGG; *Fbln5*, F-GTGTGTGGATGTGGACGAGT and R-TACCCTCCTTCCGTGTTGAT; *Rtbdn*, F-TACAGCCCACTAGGGCCTTAACTC and R-TACAGTACCGCGGAGATGGAGAT; *Lipg*, F-TGCAACAGCCAAAACCTTCT and R-TGTCCCACTTTCCTCGTGTT; and *β-actin*, F-TGGAATCCTGTGGCATCCATGAAAC and R-TAAAACGCAGCTCAGTAACAGTAACAGTCCG. 

### 4.4. Whole Transcriptome Sequencing

For the whole transcriptome sequencing of each hypothalamus extracted from the fed and fasted mice (*n* = 3, respectively), double-stranded cDNA libraries were prepared from a total of 1 μg of RNA molecules using the TruSeq Stranded mRNA Sample Prep Kit (Illumina, San Diego, CA, USA). The quality and quantity of the cDNA libraries were evaluated with the Agilent 2100 BioAnalyzer (Agilent, Santa Clara, CA, USA) and the KAPA library quantification kit (Kapa Biosystems, Wilmington, MA, USA). By using Illumina Novaseq6000 (Illumina), paired-end sequencing (2 × 100 base pairs) was carried out. 

For the whole transcriptome sequencing of each hippocampus extracted from the fed and fasted mice (*n* = 3, respectively), Quant-IT RiboGreen (Invitrogen) was used to assess the total RNA concentration and TapeStation RNA ScreenTape (Agilent) was run to estimate the integrity of the total RNA. A library with LIN greater than 7.0 was equipped using the Illumina TruSeq Stranded mRNA Sample Prep Kit (Illumina) in 1ug of total RNA for each sample. The quantity of the libraries was assessed using KAPA Library Quantification kits for Illumina Sequencing platforms according to the qPCR Quantification Protocol Guide (Kapa Biosystems), and the quality of the libraries was assessed using the TapeStation D1000 ScreenTape (Agilent). An Illumina NovaSeq (Illumina) was used for paired-end sequencing (2 × 100 bp). 

### 4.5. Whole Transcriptome Analysis

Sequence quality was assessed by FastQC v0.11.9. Trimming of low-quality and adapter sequences was performed by cutadapt v3.4 [36] and Trimmomatic v0.36 [37] for the hypothalamus and hippocampus, respectively. Sequences were mapped by STAR v.2.7.3a [38], using the 2-pass method–to–mouse reference genome from the Ensembl database (GRCm38, 101 released). Assigning sequence reads to genes was carried out by the featureCounts [39] algorithm, which is a suitable program for reading the summarization from RNA-sequencing experiments. 

### 4.6. Differential Expression Genes Analysis, Secretory Genes Analysis, and Pathway Analysis

The EdgeR v3.32.1 [40] R package was used to estimate DEGs from RNA-sequencing data based on the over-dispersed Poisson model and Empirical Bayes methods. The DEG analysis was conducted with the glmQLFit/glmQLFTest function of edgeR, which applied the quasi-likelihood F-test method in fasted mice compared to fed mice. The FDR and the log2FC were extracted. FDR was calculated using the Benjamini–Hochberg method. DEGs results were visualized with a volcano plot and heatmap using calibrate v1.7.7 and ggplot2 v3.3.5 in the R package. 

MetazSecKB [19], secretome and proteome knowledgebase of the human and animal, was used to get lists of “curated secreted” proteins and “high likely” secreted proteins. “Curated secreted” proteins are the reviewed dataset to be ‘secreted’ or ‘extracellular’ from UniProtKB/Swiss-Prot, and “high likely” secreted proteins are a dataset called to be secreted or a secretory signal peptide predicted from four algorithms, SignalP4, Phobius, TargetP, and WoLF PSORT7. Two protein datasets were converted to the gene ID of the Ensembl using the biomaRt [41] v2.46.3 R package, the mapping tools for the incorporation of a genomic dataset. Only genes with FDR less than 0.05 and absolute log2FC greater than 1.0 were used. Raw counts of feature Counts’ output were transformed to TPM (Transcripts Per Million) to visualize the heatmap of the mean of expression level. The Seaborn [42] v0.11.1 python library was used for visualization. 

Enriched function annotation and canonical pathway analysis (generalized pathways that represent common properties of a particular signaling module or pathway) were performed using IPA software (Ingenuity Systems, Redwood City, CA, USA).

### 4.7. Statistical Analysis

Statistical analyses were performed using the Prism 9.0 software (GraphPad Software, San Diego, CA, USA). All the data are expressed as the means ± SEMs. Unpaired two-tail Student’s *t*-test was performed to analyze the significance between the two experimental groups. *p* values < 0.05 were considered statistically significant.

## Figures and Tables

**Table 1:** The 32 differentially expressed genes (DEGs) in only hypothalamus (FDR ≤ 0.05 and abs(log2FC) > 1.5 or secretory-related genes). Secretory-related genes in the Metazoa Human/Animal Secretome and Subcellular Proteome Knowledge Base (MetazSecKB) or DEGs with an absolute log2 fold change larger than 1.5 and an FDR less than 0.05 are chosen. Secretory-related genes are indicated in bold.

Gene	Description	log2FC	*p*-Value	FDR
*Phyhd1*	phytanoyl-CoA dioxygenase domain containing 1	2.01	1.10 × 10^−6^	1.47 × 10^−3^
***Fbln5***	**fibulin 5**	**1.51**	**1.70 × 10^−6^**	**1.54 × 10^−3^**
*Acss3*	acyl-CoA synthetase short chain family member 3	1.74	2.81 × 10^−6^	1.61 × 10^−3^
***Wfikkn2***	**WAP, follistatin/kazal, immunoglobulin, kunitz and netrin domain containing 2**	**2.73**	**2.43 × 10^−6^**	**1.61 × 10^−3^**
***Col5a3***	**collagen type V alpha 3 chain**	**1.14**	**4.44 × 10^−6^**	**1.80 × 10^−3^**
*Itgad*	integrin subunit alpha D	2.94	4.65 × 10^−6^	1.83 × 10^−3^
*Slc38a5*	solute carrier family 38 member 5	−2.02	9.71 × 10^−6^	2.56 × 10^−3^
*Arhgef37*	Rho guanine nucleotide exchange factor 37	1.63	1.14 × 10^−5^	2.65 × 10^−3^
*A730020M07Rik*	RIKEN cDNA A730020M07 gene	1.84	1.16 × 10^−5^	2.67 × 10^−3^
*Pla2g3*	phospholipase A2 group III	2.87	2.41 × 10^−5^	4.21 × 10^−3^
*Hr*	HR lysine demethylase and nuclear receptor corepressor	1.84	2.87 × 10^−5^	4.41 × 10^−3^
*Stab2*	stabilin 2	1.79	4.20 × 10^−5^	5.57 × 10^−3^
*5330429C05Rik*	RIKEN cDNA 5330429C05 gene	3.09	1.15 × 10^−4^	1.12 × 10^−2^
*Uhrf1*	ubiquitin like with PHD and ring finger domains 1	−1.52	1.22 × 10^−4^	1.16 × 10^−2^
*Ctla2a*	cytotoxic T lymphocyte-associated protein 2 alpha	1.57	1.30 × 10^−4^	1.19 × 10^−2^
*Gabrr2*	gamma-aminobutyric acid type A receptor subunit rho2	1.90	1.50 × 10^−4^	1.28 × 10^−2^
*Hsf3*	heat shock transcription factor 3	3.21	1.84 × 10^−4^	1.44 × 10^−2^
*Pcdh12*	protocadherin 12	−1.80	3.05 × 10^−4^	1.91 × 10^−2^
*Aplnr*	apelin receptor	−1.57	3.91 × 10^−4^	2.15 × 10^−2^
*Sds*	serine dehydratase	1.63	4.00 × 10^−4^	2.17 × 10^−2^
*Tmem82*	transmembrane protein 82	2.18	4.23 × 10^−4^	2.25 × 10^−2^
*Map3k6*	mitogen-activated protein kinase kinase kinase 6	2.01	4.63 × 10^−4^	2.33 × 10^−2^
*Cd300lf*	CD300 molecule like family member f	2.07	5.76 × 10^−4^	2.63 × 10^−2^
***Lipg***	**lipase G, endothelial type**	**−1.26**	**5.80 × 10^−4^**	**2.64 × 10^−2^**
***Pglyrp1***	**peptidoglycan recognition protein 1**	**1.08**	**8.66 × 10^−4^**	**3.39 × 10^−2^**
***Rtbdn***	**retbindin**	**1.17**	**1.02 × 10^−3^**	**3.76 × 10^−2^**
*Myh1*	myosin heavy chain 1	1.54	1.05 × 10^−3^	3.79 × 10^−2^
*Gkn3*	gastrokine 3	−1.69	1.21 × 10^−3^	4.14 × 10^−2^
*Ankrd63*	ankyrin repeat domain 63	−1.61	1.62 × 10^−3^	4.78 × 10^−2^
*Klrg2*	killer cell lectin like receptor G2	1.88	1.67 × 10^−3^	4.89 × 10^−2^
*Fyb2*	FYN binding protein 2	1.83	1.73 × 10^−3^	5.01 × 10^−2^
***Agrp***	**agouti related neuropeptide**	**1.24**	**1.98 × 10^−3^**	**5.38 × 10^−2^**

Abbreviations: abs(log2FC), absolute log2 fold change; FDR, false discovery rate.

**Table 2:** Top 20 enriched functional annotations of differentially expressed genes in fasted mice compared with those of fed mice. The top 20 of 199 (*p* < 0.05) in enriched functional annotation categories are exhibited. Ingenuity pathway analysis software (IPA) was used to analyze enriched functional annotations with an input of 70 DEGs in only the hypothalamus (FDR ≤ 0.05 and abs(log2FC) > 1.0).

Functional Annotation	*p*-Value	Genes
Ingestion by mice	2.03 × 10^−4^	*AGRP*, *AGTR1*, *APLNR*, *C8orf44-SGK3/SGK3*, *MYH1*, *NPY*
Quantity of lysophosphatidylcholine	1.75 × 10^−3^	*MFSD2A*, *PLA2G3*
Hyperphagia	2.07 × 10^−3^	*AGRP*, *AGTR1*, *NPY*
Formation of actin bundles	2.14 × 10^−3^	*AIF1*, *FAM107A*
Appetite	2.56 × 10^−3^	*AGRP*, *NPY*
Function of vascular endothelial cells	2.79 × 10^−3^	*LIPG*, *MFSD2A*
Abnormal skeletal muscle fiber type ratio	3.03 × 10^−3^	*MYH1*, *WFIKKN2*
Cutis laxa	3.28 × 10^−3^	*FBLN5*
Transmembrane potential of mitochondrial outer membrane	3.28 × 10^−3^	*HR*
Demyelination of cervical spinal cord	3.28 × 10^−3^	*CD300LF*
Organization of elastic fibers	3.28 × 10^−3^	*FBLN5*
Activation of BMMC cells	3.28 × 10^−3^	*CD300LF*
Internalization of phospholipid	3.28 × 10^−3^	*MFSD2A*
Elastinopathy	3.28 × 10^−3^	*FBLN5*
Transport of lysophosphatidic acid	3.28 × 10^−3^	*MFSD2A*
Hydrolysis of 1-oleoyl lysophosphatidylcholine	3.28 × 10^−3^	*PNPLA7*
Development of infundibulum of hair follicle	3.28 × 10^−3^	*HR*
Infection by Norovirus	3.28 × 10^−3^	*CD300LF*
Obesity of epididymal fat	3.28 × 10^−3^	*AGTR1*
Synthesis of neointima	3.28 × 10^−3^	*AGTR1*

Abbreviation: BMMC, bone marrow mononuclear cells.

**Table 3:** Top 16 canonical pathways of differentially expressed genes in fasted mice compared with those of fed mice. The top 16 (*p* < 0.05) results of canonical pathway analyses are listed. Canonical pathways are identified with an input of 70 DEGs in only the hypothalamus (FDR ≤ 0.05 and abs(log2FC) > 1.0) using IPA. The number of genes in the input divided by the number of genes involved in the identified pathway is referred to as the ratio. To determine the probability that the association between the 70 genes and canonical pathway can be explained solely by chance, the Fisher exact test was used to calculate the *p*-value. The logarithmic value (−log[*p*-value]) and two digits to the right of the decimal point were used to indicate the *p*-value.

Ingenuity Canonical Pathways	−log(*p*-Value)	Ratio	Molecules
Apelin Cardiac Fibroblast Signaling Pathway	2.59	0.087	AGTR1, APLNR
Apelin Liver Signaling Pathway	2.49	0.077	APLNR, COL5A3
Thiosulfate Disproportionation III (Rhodanese)	2.01	0.333	TST
L-serine Degradation	2.01	0.333	SDS
Acetate Conversion to Acetyl-CoA	1.88	0.250	ACSS3
Triacylglycerol Degradation	1.87	0.037	LIPG, PNPLA7
Protein Citrullination	1.79	0.200	PADI2
Phospholipases	1.75	0.032	LIPG, PLA2G3
Gustation Pathway	1.63	0.016	GABRR2, LIPG, PKD2L1
Hepatic Fibrosis/Hepatic Stellate Cell Activation	1.63	0.016	AGTR1, COL5A3, MYH1
Leptin Signaling in Obesity	1.60	0.027	AGRP, NPY
VDR/RXR Activation	1.57	0.026	HR, SERPINB1
RHOGDI Signaling	1.51	0.014	CDH19, ITGAD, MYH1
Glycine Betaine Degradation	1.49	0.100	SDS
Ketogenesis	1.45	0.091	HMGCS2
Mevalonate Pathway I	1.38	0.077	HMGCS2

Abbreviations: VDR, Vitamin D receptor; RXR, retinoid X receptor; RHOGDI, Rho GDP-dissociation inhibitor.

## Data Availability

All data reported in the manuscript.

## Supplementary Materials

The supporting information can be downloaded at: https://www.mdpi.com/article/10.3390/ijms24043204/s1.
