# Supplementary material for: Whole Transcriptome Analysis of Hypothalamus in Mice during Short-Term Starvation
Source: Int J Mol Sci. 2023 Feb 6;24(4):3204. doi: 10.3390/ijms24043204 (PMC9968171; doi:10.3390/ijms24043204)
Supplement: Supplementary file 1 [file ijms-24-03204-s001.zip › ijms-2095715-supplementary.pdf]

Supplementary Data

Gene	Description	log2FC	P-value	FDR
Fam107a	family with sequence similarity 107 member A	1.08	4.05E-08	0.0002
Hmgcs2	3-hydroxy-3-methylglutaryl-CoA synthase 2	1.20	2.13E-07	0.001
Tst	thiosulfate sulfurtransferase	-1.27	8.52E-07	0.001
Padi2	peptidyl arginine deiminase 2	-1.10	1.44E-06	0.001
Phyhd1	phytanoyl-CoA dioxygenase domain containing 1	2.01	1.10E-06	0.001
Fbln5	fibulin 5	1.51	1.70E-06	0.002
Mfsd2a	major facilitator superfamily domain containing 2A	1.42	3.08E-06	0.002
Acss3	acyl-CoA synthetase short chain family member 3	1.74	2.81E-06	0.002
Wfikkn2	WAP, follistatin/kazal, immunoglobulin, kunitz and netrin domain containing 2	2.73	2.43E-06	0.002
C030029H02Rik	RIKEN cDNA C030029H02 gene	-1.23	4.13E-06	0.002
Col5a3	collagen type V alpha 3 chain	1.14	4.44E-06	0.002
Itgad	integrin subunit alpha D	2.94	4.65E-06	0.002
Ugt8a	UDP galactosyltransferase 8A	-1.03	5.52E-06	0.002
Gbp3	guanylate binding protein 3	-1.15	8.27E-06	0.002
Pnpla7	patatin like phospholipase domain containing 7	1.23	7.78E-06	0.002
Slc38a5	solute carrier family 38 member 5	-2.02	9.71E-06	0.003
Cdh19	cadherin 19	1.19	9.81E-06	0.003
Alpl	alkaline phosphatase, biomineralization associated	-1.44	1.03E-05	0.003
Serpinb1a	uncharacterized LOC105262837	-1.10	1.02E-05	0.003
Arhgef37	Rho guanine nucleotide exchange factor 37	1.63	1.14E-05	0.003
A730020M07Rik	RIKEN cDNA A730020M07 gene	1.84	1.16E-05	0.003
Pkd2l1	polycystin 2 like 1, transient receptor potential cation channel	-1.11	1.74E-05	0.003
Rab7b	RAB7B, member RAS oncogene family	-1.16	1.82E-05	0.004
Pla2g3	phospholipase A2 group III	2.87	2.41E-05	0.004
Hr	HR lysine demethylase and nuclear receptor corepressor	1.84	2.87E-05	0.004
Aif1	allograft inflammatory factor 1	-1.26	3.42E-05	0.005
Npy	neuropeptide Y	1.39	3.43E-05	0.005
Stab2	stabilin 2	1.79	4.20E-05	0.006
Angptl2	angiopoietin like 2	-1.26	6.21E-05	0.007
5330429C05Rik	RIKEN cDNA 5330429C05 gene	3.09	1.15E-04	0.011
Uhrf1	ubiquitin like with PHD and ring finger domains 1	-1.52	1.22E-04	0.012
Fam83d	family with sequence similarity 83 member D	1.44	1.31E-04	0.012
Ctla2a	cytotoxic T lymphocyte-associated protein 2 alpha	1.57	1.30E-04	0.012
Gabrr2	gamma-aminobutyric acid type A receptor subunit rho2	1.90	1.50E-04	0.013
Hsf3	heat shock transcription factor 3	3.21	1.84E-04	0.014
Agtr1a	angiotensin II receptor, type 1a	1.12	2.16E-04	0.016
Rnase1	ribonuclease A family member 1, pancreatic	-1.23	3.01E-04	0.019
Pcdh12	protocadherin 12	-1.80	3.05E-04	0.019
Aplnr	apelin receptor	-1.57	3.91E-04	0.021
Lrrc29	leucine rich repeat containing 29	1.03	3.86E-04	0.021

Sds	serine dehydratase	1.63	4.00E-04	0.022
Fmo2	flavin containing dimethylaniline monoxygenase 2	1.49	4.15E-04	0.022
Cd163	CD163 molecule	1.10	4.24E-04	0.023
Tmem82	transmembrane protein 82	2.18	4.23E-04	0.023
Gipc3	GIPC PDZ domain containing family member 3	-1.10	4.49E-04	0.023
Dnah14	dynein axonemal heavy chain 14	1.21	4.64E-04	0.023
Map3k6	mitogen-activated protein kinase kinase kinase 6	2.01	4.63E-04	0.023
Cd300lf	CD300 molecule like family member f	2.07	5.76E-04	0.026
Epx	eosinophil peroxidase	-1.41	5.80E-04	0.026
Lipg	lipase G, endothelial type	-1.26	5.80E-04	0.026
Cd180	CD180 molecule	-1.05	7.96E-04	0.032
Pglyrp1	peptidoglycan recognition protein 1	1.08	8.66E-04	0.034
Gm15201	predicted gene 15201	1.11	9.11E-04	0.034
Rtbdn	retbindin	1.17	1.02E-03	0.038
Slc18a1	solute carrier family 18 member A1	1.37	1.03E-03	0.038
Myh1	myosin heavy chain 1	1.54	1.05E-03	0.038
Slc43a3	solute carrier family 43 member 3	1.33	1.09E-03	0.039
Anxa8	annexin A8	1.01	1.16E-03	0.040
Gkn3	gastrokine 3	-1.69	1.21E-03	0.041
Fut9	fucosyltransferase 9	1.10	1.26E-03	0.042
A230009B12Rik	RIKEN cDNA A230009B12 gene	-1.33	1.29E-03	0.043
Neurod6	neuronal differentiation 6	1.04	1.34E-03	0.043
Srarp	steroid receptor associated and regulated protein	-1.02	1.38E-03	0.044
Gm29508	predicted gene 29508	-1.09	1.48E-03	0.045
Ankrd63	ankyrin repeat domain 63	-1.61	1.62E-03	0.048
Klrg2	killer cell lectin like receptor G2	1.88	1.67E-03	0.049
Fyb2	FYN binding protein 2	1.83	1.73E-03	0.050
Sgk3	serum/glucocorticoid regulated kinase family member 3	1.41	1.83E-03	0.052
Fkbp5	FKBP prolyl isomerase 5	1.20	1.86E-03	0.052
Agrp	agouti related neuropeptide	1.24	1.98E-03	0.054

Supplementary Table S1. Differentially Expressed Genes in Hypothalamus only (FDR ≤ 0.05 and abs(log2FC) > 1.0). 70 differentially expressed genes of the hypothalamus with an absolute log2 fold change larger than 1.0 and an FDR less than 0.05 except for 11 genes that are commonly significantly expressed in the hypothalamus and hippocampus are shown. Abbreviations: abs(log2FC), absolute log2 fold change; FDR, false discovery rate.

Gene	Description	log2FC	P-value	FDR
Rtp1	Receptor Transporter Protein 1	2.26	5.14.E-06	0.006
Etnppl	Ethanolamine-Phosphate Phospho-Lyase	1.48	1.35.E-05	0.011
Lcn2	Lipocalin 2	2.93	1.24.E-05	0.011
Ada	Adenosine Deaminase	1.54	2.78.E-05	0.014
COX3	Mitochondrially Encoded Cytochrome C Oxidase III	2.22	3.02.E-05	0.014
Atp2c2	ATPase Secretory Pathway Ca2+ Transporting 2	1.22	3.36.E-05	0.015
Plekhf1	Pleckstrin Homology And FYVE Domain Containing 1	1.05	3.64.E-05	0.016
Gbp5	Guanylate Binding Protein 5	-1.31	9.36.E-05	0.026
Tmem52	Transmembrane Protein 52	1.33	2.22.E-04	0.041
H2bc6	H2B Clustered Histone 6	-1.05	4.17.E-04	0.050

Supplementary Table S2. Differentially Expressed Genes in Hippocampus only (FDR ≤ 0.05 and abs(log2FC) > 1.0). 10 differentially expressed genes of the hippocampus with an absolute log2 fold change larger than 1.0 and an FDR less than 0.05 except for 10 genes that are commonly significantly expressed in hypothalamus and hippocampus are shown.
Abbreviations: abs(log2FC), absolute log2 fold change; FDR, false discovery rate.

Gene Name	log2FC of Hypothalamus	FDR of Hypothalamus	log2FC of Hippocampus	FDR of Hippocampus
Tnc	-1.07	0.003	-1.12	0.011
Angptl4	1.30	0.004	1.32	0.005
Tekt4	1.33	0.016	1.27	0.030
Xdh	1.88	0.031	1.15	0.043
Kirrel2	1.88	0.007	1.02	0.002
Cdkn1a	2.34	0.006	1.65	0.008
Sult1a1	2.40	0.000	1.47	0.001
Hif3a	2.66	0.004	1.75	0.001
Otoa	3.02	0.002	2.52	0.043
Lao1	3.53	0.002	2.44	0.053
Plin4	4.37	0.002	3.21	0.011

Supplementary Table S3. Differentially Expressed Genes in both Hypothalamus and Hippocampus (FDR ≤ 0.05 and abs(log2FC) > 1.0). 11 common differentially expressed genes in both the hypothalamus and hippocampus with an absolute log2 fold change larger than 1.0 and an FDR less than 0.05 are shown.
Abbreviations: abs(log2FC), absolute log2 fold change; FDR, false discovery rate.
